# Evaluation of Tool Wear Characteristics and Machining Performance During Longitudinal–Torsional Ultrasonic Vibration Drilling of Al/Ti Stacks

**DOI:** 10.3390/mi17020227

**Published:** 2026-02-10

**Authors:** Zhaoju Zhu, Shiying Geng, Wenrong Zhu, Guang Ouyang, Yiping Huang

**Affiliations:** School of Mechanical Engineering and Automation, Fuzhou University, Fuzhou 350116, China; g1337992247@163.com (S.G.); 17268683378@163.com (W.Z.); oyg173@163.com (G.O.); huangyiping0509@outlook.com (Y.H.)

**Keywords:** tool wear, longitudinal–torsional ultrasonic vibration drilling, Al/Ti stacks, surface quality

## Abstract

Al/Ti stacks are widely used in aerospace manufacturing due to their heterogeneous and multi-property material characteristics. However, during integrated hole-making processes, the significant differences in material properties often induce abrupt variations in cutting force, leading to uneven loading along the cutting edge and non-uniform tool wear. These issues complicate the drilling process and severely hinder the advancement of manufacturing and assembly technologies for aerospace components. To address these issues, longitudinal–torsional ultrasonic vibration drilling (LTUVD) is implemented in drilling of Al/Ti stacks, which superimposes high-frequency axial and tangential vibrations onto conventional drilling, enabling a spatial elliptical cutting trajectory and periodic material separation. A spatial kinematic model of LTUVD is developed to analyze the effects of key parameters on the tool motion trajectory and chip variations. Drilling experiments are conducted on Al/Ti stacks at a defined cutting condition (30 m/min, 0.1 mm/rev) to compare the performance of conventional drilling (CD), ultrasonic vibration-assisted drilling (UVAD), and LTUVD under various conditions. The results show that LTUVD can significantly outperform the other two methods in reducing thrust force, chip breaking (especially in the titanium layer), mitigating tool wear, and improving hole wall surface quality. In addition, scanning electron microscopy (SEM) and energy-dispersive spectroscopy (EDS) analyses further reveal that LTUVD can effectively suppress thermal and adhesive wear, thereby extending tool life.

## 1. Introduction

Tool wear remains a critical challenge in drilling of laminated materials, where abrupt transitions in material properties induce severe thermo-mechanical loads, accelerating tool degradation and compromising machining quality. The evolution of tool wear directly induces sudden changes in cutting force [1,2] and deterioration in machining quality [3]. In essence, cutting force and surface integrity are dynamic responses to tool wear. Focusing solely on reducing cutting force may compromise efficiency, while optimizing surface quality alone may sacrifice sustainability. Studies on tool wear have revealed complex wear mechanisms in layered structures, including abrasive, adhesive, and diffusion wear. In CFRP/Al [4], CFRP/Ti [5], CFRP/Ti/Al [6], and Al/Ti [7] configurations, the CFRP layer tends to cause micro-chipping at the cutting edge, the aluminum layer often forms an adhesive layer on the tool surface [8], and the titanium layer, due to its low thermal conductivity and high adhesion tendency, becomes the primary source of wear, accelerating tool degradation [9]. Numerous studies have shown that PCD and diamond-coated tools exhibit enhanced wear resistance; however, under high-temperature conditions, uncoated carbide tools can offer better stability [10,11,12]. In terms of tool structure and auxiliary technologies, the use of novel step drills [13], low-frequency vibration-assisted drilling (LFVAD) [14], and forced cryogenic cooling [15] have been shown to improve chip evacuation, reduce tool load and frictional heat, significantly slow wear progression, and extend tool life. Some of these approaches have achieved tool life improvements exceeding 20% [16,17,18].

However, existing methods have inherent limitations, as conventional axial ultrasonic machining offers limited control over material failure in the cutting zone. In this study, longitudinal–torsional ultrasonic vibration drilling (LTUVD) has been introduced as a novel hybrid vibration technique. By simultaneously applying high-frequency axial and torsional vibrations, it can effectively alter the cutting trajectory, concentrate stress and energy, and enhance the impact and fracture effect of the cutting edge on the material. This leads to reduced cutting forces, lower heat generation, and improved chip evacuation, demonstrating great potential for high-quality and high-efficiency machining of stacked structures [19,20]. Ma et al. investigated the influence of amplitude ratio on the performance of longitudinal–torsional ultrasonic vibration drilling [21]. Wang et al. investigated the evolution of drill wear characteristics with the number of holes in LTUVD and the corresponding relationship between different drilling morphologies and tool wear [22]. Zhou et al. examined how interfacial temperature in LTUVD affects hole-quality characteristics [23].

Despite the significant potential of LTUVD, key challenges remain in understanding tool wear mechanisms during Al/Ti layered drilling. Currently, there is a lack of systematic analysis on the cross-scale coupling among machining parameters, cutting force response, material removal mechanisms, and abnormal tool wear evolution. Moreover, most existing studies are concentrated in the field of milling, while the interfacial material removal mechanisms and wear evolution pathways of tools under LTUVD remain unclear. To address this issue, the present study conducts a comparative investigation of CD, UVAD, and LTUVD in Al/Ti stack drilling. The analysis first examines the kinematic characteristics of each drilling method to interpret differences in chip formation behavior. Based on the force response—especially the evolution of thrust force during successive holes—the wear progression stages are identified and correlated with SEM observations and elemental analysis. Finally, the evolution of tool wear and machined surface integrity is comparatively evaluated across drilling methods as a function of hole number. Through this structured analysis, the study elucidates the mechanism by which LTUVD suppresses force growth and stabilizes tool degradation during multi-hole drilling.

## 2. Materials and Methods

The experimental materials—aluminum alloy 7075-T7451 (upper layer) and titanium alloy Ti-6Al-4V (lower layer), whose key properties are listed in Table 1—were machined into 300 mm × 55 mm × 3 mm rectangular specimens for drilling tests. Ti-6Al-4V was selected for its high specific strength and thermal stability, which are essential for aerospace engines and load-bearing components, while aluminum alloy 7075-T7451 offers a strength-to-weight ratio and fatigue resistance suited to airframe structures such as wing spars and fuselage panels. The drilling tests utilized a Kennametal B292A04000YPL solid carbide drill (Kennametal Inc., Pittsburgh, PA, USA) with an ultrafine-grained WC-Co substrate and a TiAlN coating for improved thermal and oxidation resistance. The tool, which incorporated internal cooling channels, had a diameter of 4 mm, a point angle of 140°, and a helix angle of 30°.

**Figure 1:**
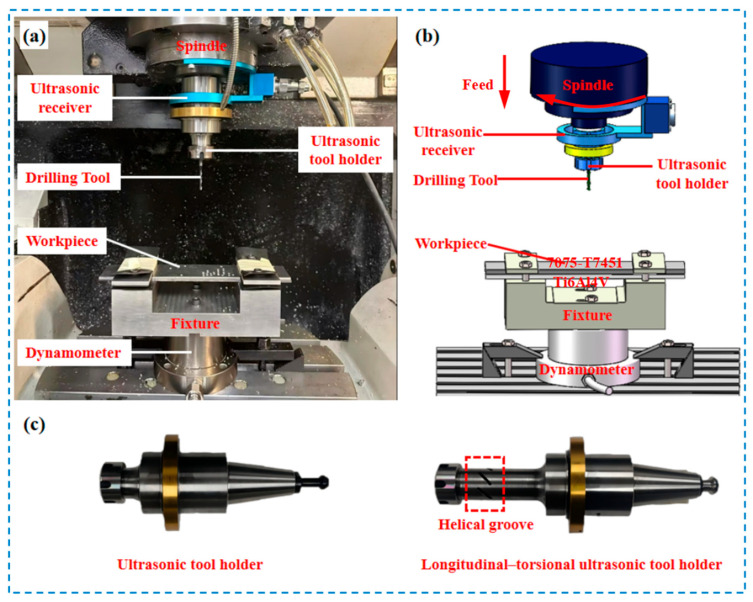
Experimental setup diagram. (**a**) Experimental setup; (**b**) Schematic diagram of the experimental setup; (**c**) Tool holder.

Machining experiments were conducted on a Mazak VARIAXIS 500-5X II 5-axis machining center (Yamazaki Mazak Corporation, Aichi-ken, Japan) (Figure 1). The workpiece fixture was rigidly mounted on a Haonai HR-F3108 four-component dynamometer (Haonai Instrument Co., Ltd., Jinan, China) (1 kHz sampling rate), itself secured to the machine table. Both longitudinal–torsional and conventional ultrasonic systems (Super sonics YC2050A series, Dongguan Supersonics Precision Technology Co., Ltd., Dongguan, China) used HSK32E tool holders (Super sonics YC2050A series, Dongguan Supersonics Precision Technology Co., Ltd., Dongguan, China) operating at 20 kHz. For the longitudinal–torsional system, a phase difference in π/2 between the longitudinal and torsional vibrations was employed. This value is inherently defined by the resonant design of the ultrasonic horn, which ensures stable, synchronized vibration output and generates the spatial elliptical tool trajectory central to the LTUVD mechanism. An integrated ultrasonic receiver performed automatic frequency calibration upon tool holder reinstallation to maintain resonance, activating programmed vibrations through embedded piezoelectric transducers upon signal synchronization. Experimental parameters (Table 2) were determined through literature review, theoretical modeling, and preliminary tests. The selected cutting speed and feed rate fall within the commonly reported range for Al/Ti stack drilling and were confirmed through preliminary trials to ensure stable cutting and clear differentiation of the vibration-assisted mechanisms. All trials used new tools from the same batch, with triplicate repetitions to ensure statistical validity.

Surface morphology and microstructure were characterized using a Veeo Contour GT-K1 3D profilometer (Veeo Instruments Co., Ltd., Suzhou, China). Tool wear analysis was performed with an FEI Quanta 250 SEM (FEI Company, Hillsboro, OR 97124, USA) equipped with EDS, enabling detailed cutting-edge morphology examination and elemental mapping under different machining conditions. Phase evolution near the hole wall was assessed by X-ray diffraction (XRD) across various machining methods and hole counts, with particular focus on the transformation behavior of the α/β phase ratio in the titanium alloy.

## 3. Results

### 3.1. Mechanistic Study of Longitudinal–Torsional Ultrasonic Vibration Drilling

**Figure 2:**
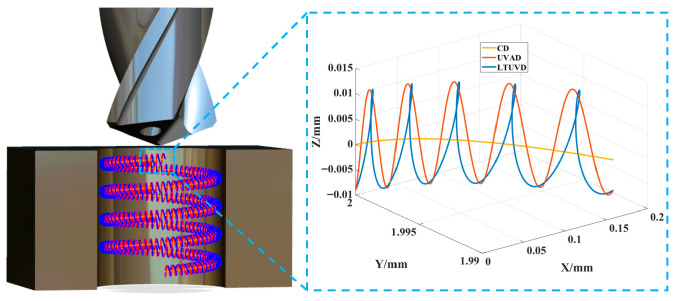
Comparison of motion trajectories for different machining methods.

**Figure 3:**
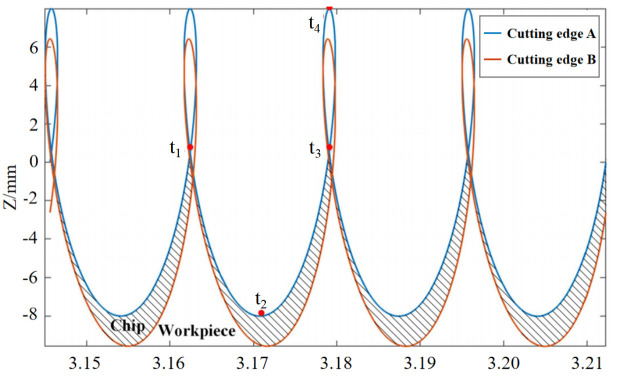
The Z-axis displacement of intermittent LTUVD.

LTUVD involves subjecting the drill bit to a longitudinal ultrasonic vibration while simultaneously applying a torsional ultrasonic vibration sharing the same frequency but possessing a specific phase difference relative to the longitudinal vibrations in its tangential direction. Consequently, points on the tool’s cutting edge experience coupled longitudinal–torsional vibrations, producing an elliptical cutting trajectory in two-dimensional (2D) and three-dimensional (3D) space, as shown in Figure 2 and Figure 3. This mode fundamentally differs from conventional drilling (CD), which is a continuous cutting process, and axial ultrasonic-vibration assisted drilling (UVAD), which employs only axial vibration to create an intermittent, linear-separation cutting action. In contrast, LTUVD’s synchronized axial–torsional vibrations generate a complex 3D elliptical trajectory, leading to enhanced chip segmentation and more effective tool-workpiece separation. The 2D and 3D tool motion trajectory of a point located at the outermost edge of the cutting edge during LTUVD can be expressed by the following equations:

z(t) = −f_r_t/60 + A _1_ sin2πft

(1) x(t) = rsin(2πnt/60 + (A _2_ sin(2πft + ϕ))/r)

y(t) = rcos(2πnt/60 + (A _2_ sin(2πft + ϕ))/r),

(2) z(t) = −f_r_t/60 + A _1_ sin2 π ft  s(t) = 2πnrt/60 + A _2_ cos(2πft)/r,

where s(t), r, A_1_, A_2_, f, f_r_, n, and φ are the circumferential displacement of a point on the cutting edge, drill bit radius, longitudinal amplitude, torsional amplitude, vibration frequency, feed rate, spindle speed, and phase difference between longitudinal and torsional vibrations, respectively.

**Figure 4:**
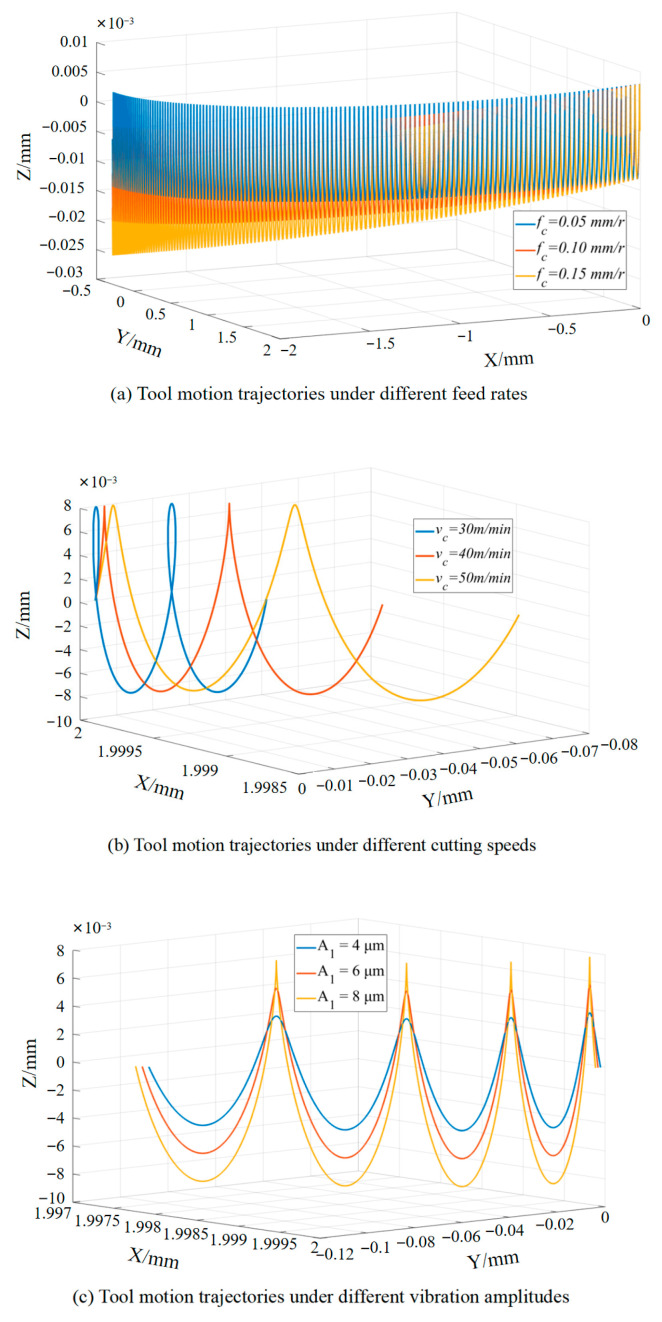
Trajectory of a point on the cutting edge in LTUVD.

The motion trajectory of LTUVD is governed by cutting parameters (feed rate and cutting speed) and vibration parameters (amplitude). As illustrated in Figure 4, MATLAB (R2023a)-generated trajectories based on the spatial kinematics of LTUVD reveal distinct pattern variations with parameter changes. In Figure 4a, increased feed rate lead to greater forward displacement per cycle and wider spacing between successive toolpaths, reducing trajectory overlap. This lower density correlates with increased surface asperities and potential deterioration of hole wall roughness. Figure 4b shows that lower cutting speeds enhance the definition of elliptical trajectories and increase path overlap, whereas higher rotational velocities diminish the relative effect of torsional vibration, thereby reducing its efficacy in modulating cutting loads and surface quality. Thus, the benefits of torsional vibration are most pronounced under low-speed conditions. As seen in Figure 4c, larger longitudinal amplitudes intensify oscillation along the Z-axis, promoting periodic tool-workpiece disengagement. This mechanism aids heat dissipation and mitigates tool wear by shortening continuous cutting time; however, it also raises localized impact stresses, creating competing effects on surface integrity through thermo-mechanical coupling [24].

Based on the kinematic analysis above, the subsequent experimental study adopts the following parameters: cutting speed of 30 m/min, feed rate of 0.1 mm/rev, and longitudinal amplitude of 6 μm.

**Figure 5:**
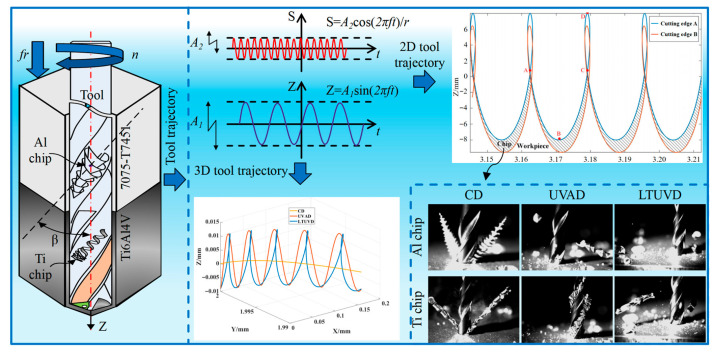
Comparison of chip morphology for different drilling methods.

Chip shape serves as a distinct indicator of tool motion trajectories and critically influences tool wear. As shown in Figure 5, while aluminum chips generally form spirals under three drilling methods. CD produces continuous spiral chips. The introduction of axial vibration in UVAD results in significantly more compact spirals that begin to fracture, whereas LTUVD directly generates short, fragmented chips due to its intense intermittent cutting action. In contrast, titanium alloy chips exhibit notable morphological differences. CD produces long, thin, tightly wound ribbons accompanied by intense smoke due to high temperatures. Although the application of axial vibration promotes chip folding, it fails to prevent tool wrapping. LTUVD, however, predominantly generates fragmented and short chips, underscoring the advantages of its intermittent cutting mechanism.

### 3.2. Evolution of Cutting Forces

#### 3.2.1. Effect of Drilling Methods on Cutting Force

**Figure 6:**
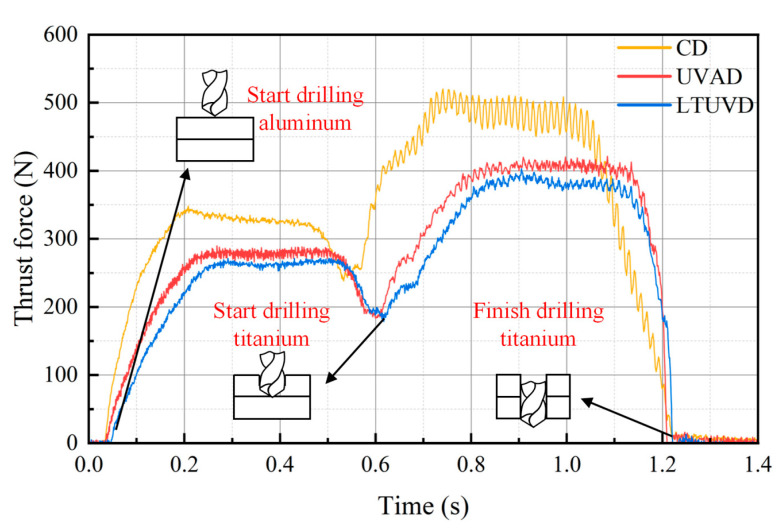
Variation in thrust force under different drilling methods.

The observed thrust force characteristics, as depicted in Figure 6, provide direct evidence for the wear-mitigation effect of LTUVD. Throughout the drilling process of the Al/Ti stack, the thrust force in LTUVD remains consistently lower than in CD and UVAD. During the drilling process, the intermittent cutting mechanism of LTUVD effectively disrupts continuous chip formation, thereby reducing resistance to material plastic deformation. More importantly, during the steady-state drilling phase, the maximum thrust force is reached in both aluminum and titanium alloy drilling. In contrast, LTUVD maintains a more stable thrust profile with lower magnitude in this critical stage—a behavior attributed to its improved chip evacuation and fragmentation capability, as evidenced by chip morphology analysis. By producing short, fragmented chips instead of long continuous ribbons, LTUVD minimizes chip–tool contact area and prevents adhesion and re-cutting. This reduction in mechanical load and frictional heat directly mitigates tool wear, leading to extended tool life and improved machining reliability.

#### 3.2.2. Effect of Tool Wear on Cutting Force

**Figure 7:**
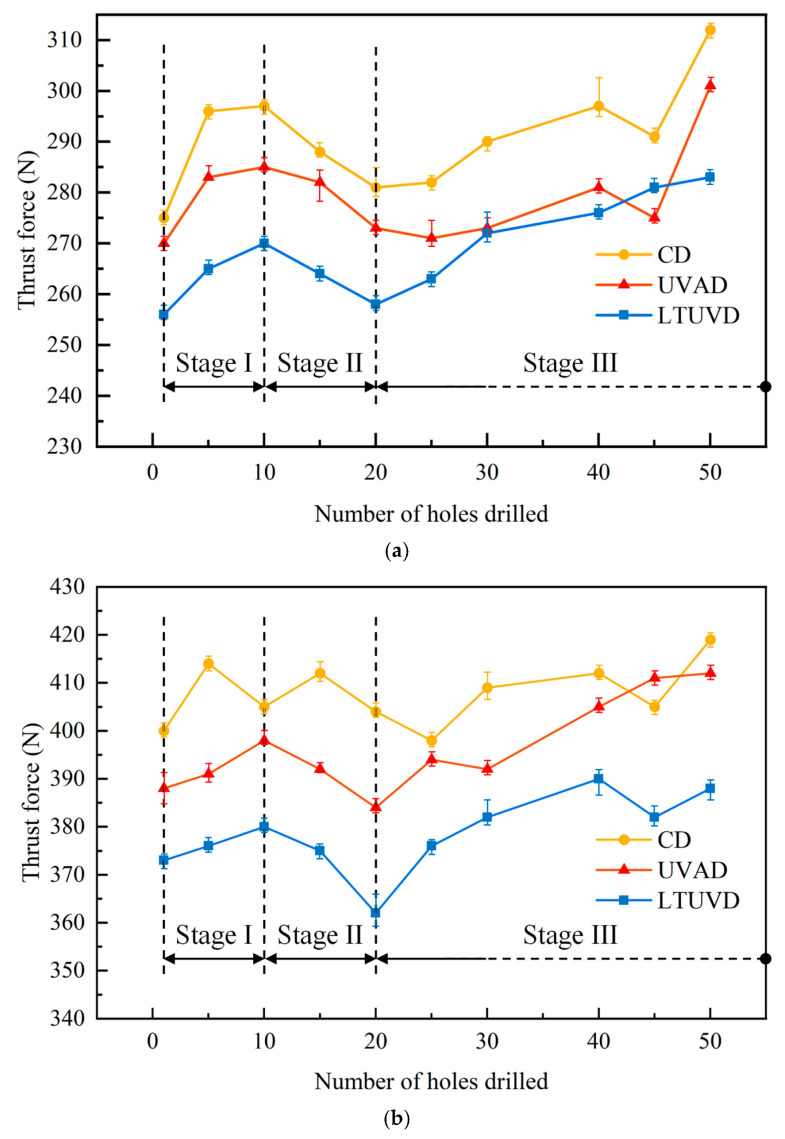
Variation in thrust force with the number of drilled holes under different drilling methods. (**a**) Evolution of thrust force in Al with the number of drilled holes; (**b**) Evolution of thrust force in Ti with the number of drilled holes.

**Figure 8:**
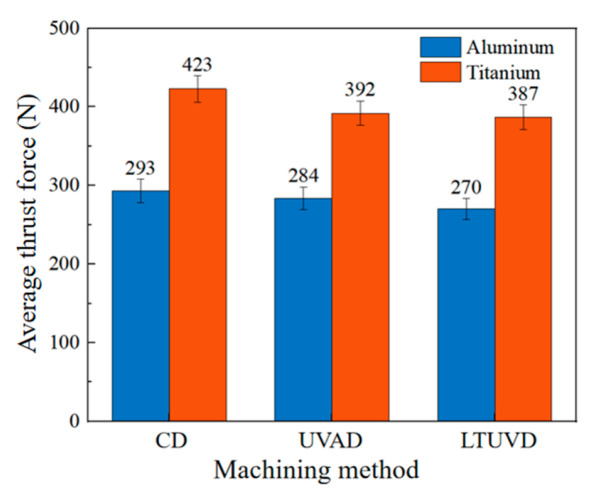
Variation in average thrust force under different drilling methods.

Figure 7 demonstrates the evolution of cutting forces with the number of drilled holes under different drilling methods. During LTUVD of Al/Ti stacks, both thrust forces exhibit a triphasic evolution: initial gradual increase, subsequent reduction, and final resurgence. In the primary phase (Holes 1st–10th), force escalation correlates with initial tool edge micro-chipping and built-up edge (BUE) formation. The intermediate phase (Holes 10th–20th) reveals force attenuation through ultrasonic-induced BUE removal and vibration-assisted chip evacuation. The terminal phase (Holes 20th–50th) shows renewed force growth due to progressive flank wear and diminished vibration transmission efficiency. Figure 7 quantifies the average thrust forces for holes 1st–30th through both material layers. Comparative analysis of Figure 7 and Figure 8 confirms that UVAD and LTUVD progressively enhance thrust force reduction efficacy across successive holes while effectively suppressing force escalation. By the 30th hole, the cumulative suppression effect reaches 18–22% for LTUVD compared with 12–15% for UVAD, indicating improved long-term stability of LTUVD (Table 3).

**Table 3:** Comparison of thrust force for CD, UVAD, and LTUVD at different hole numbers.

Drilling Method	Number of Holes	Thrust Force in Al (N)	Average Thrust Force (N)	Thrust Force in Ti (N)	Average Thrust Force (N)
CD	1	275 ± 1.3	293 ± 18	400 ± 1.4	423 ± 23
	5	295 ± 1.1		414 ± 1.2	
	10	296 ± 0.9		405 ± 1.2	
	15	288 ± 1.8		412 ± 1.8	
	20	280 ± 1.5		404 ± 1.5	
	25	282 ± 1.1		398 ± 1.4	
	30	288 ± 1.6		409 ± 2.2	
	40	294 ± 4.8		412 ± 1.4	
	45	289 ± 1.1		405 ± 1.3	
	50	310 ± 1.0		419 ± 1.3	
UVAD	1	270 ± 1.2	284 ± 14	388 ± 2.2	392 ± 20
	5	283 ± 2.2		391 ± 1.6	
	10	285 ± 1.8		398 ± 1.5	
	15	281 ± 3.8		392 ± 1.1	
	20	272 ± 1.2		384 ± 1.5	
	25	271 ± 3.0		394 ± 1.4	
	30	270 ± 2.0		392 ± 1.2	
	40	280 ± 1.8		405 ± 1.4	
	45	272 ± 1.8		409 ± 1.0	
	50	300 ± 2.0		412 ± 1.2	
LTUVD	1	256 ± 2.0	270 ± 14	373 ± 1.5	387 ± 25
	5	265 ± 1.8		376 ± 1.2	
	10	270 ± 1.6		380 ± 1.2	
	15	264 ± 1.6		375 ± 1.2	
	20	258 ± 1.8		362 ± 2.4	
	25	263 ± 1.6		376 ± 1.2	
	30	271 ± 1.4		382 ± 2.0	
	40	272 ± 1.2		390 ± 1.8	
	45	279 ± 1.8		386 ± 1.6	
	50	281 ± 1.5		393 ± 1.5	

These findings substantiate LTUVD’s capacity to maintain thrust force reduction under multi-hole processing conditions, attributable to its sustained vibration energy delivery and wear mitigation mechanisms.

### 3.3. Mechanism Study of Tool Wear

#### 3.3.1. SEM Analysis of Tool Wear

**Figure 9:**
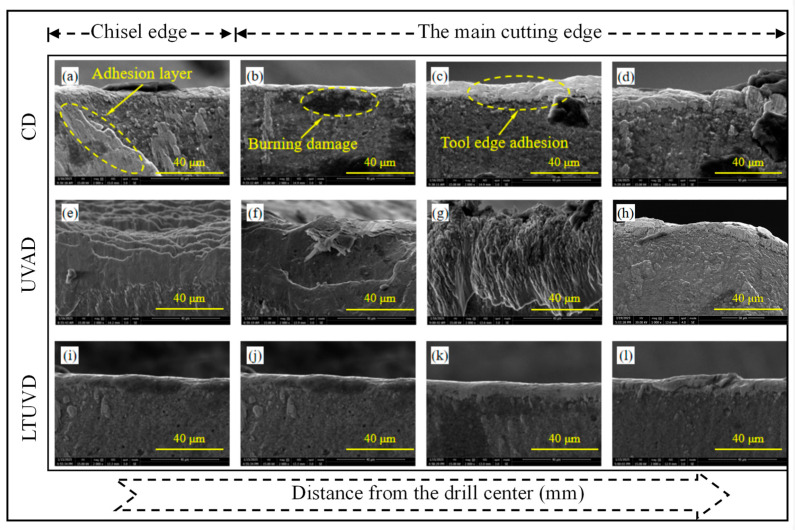
SEM images (2000×) of tool edge wear after drilling the 10th hole: (**a**–**d**) tool images under CD; (**e**–**h**) tool images under UVAD; (**i**–**l**) tool images under LTUVD.

**Figure 10:**
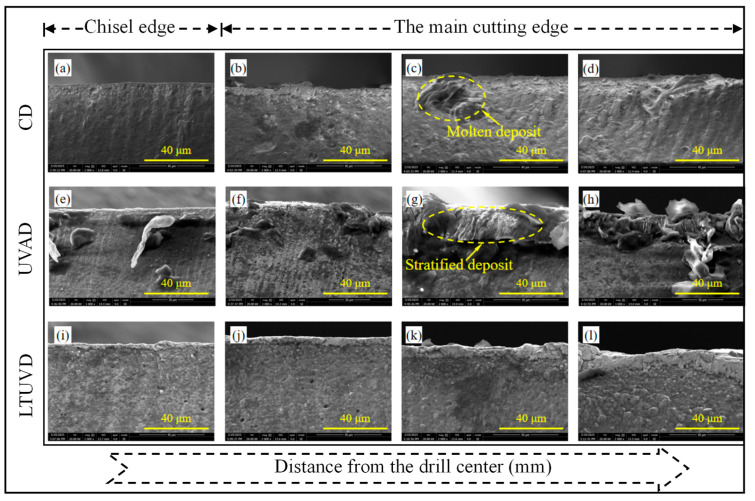
SEM images (2000×) of tool edge wear after drilling the 20th hole: (**a**–**d**) tool images under CD; (**e**–**h**) tool images under UVAD; (**i**–**l**) tool images under LTUVD.

**Figure 11:**
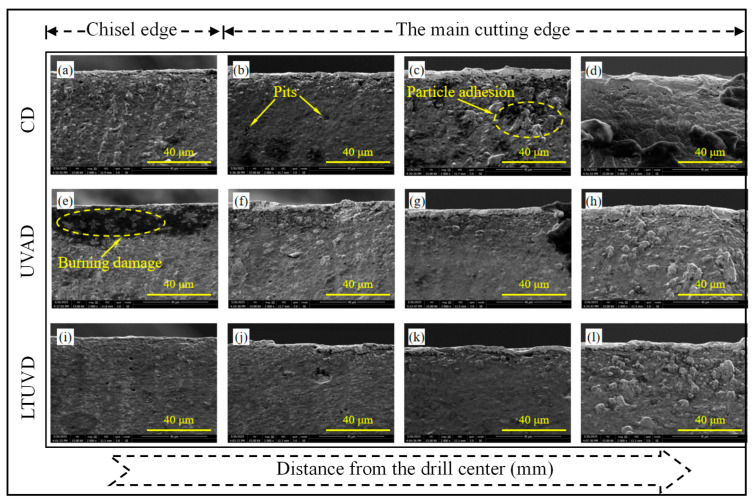
SEM images (2000×) of tool edge wear after drilling the 30th hole: (**a**–**d**) tool images under CD; (**e**–**h**) tool images under UVAD; (**i**–**l**) tool images under LTUVD.

As shown in Figure 9, Figure 10 and Figure 11, SEM was employed to systematically evaluate flank wear progression after the 10th, 20th, and 30th holes during drilling of Al/Ti stacks using CD, UVAD, and LTUVD, which reveals distinct evolutionary patterns in wear mechanisms. CD tools exhibited progressive degradation characterized by initial molten deposits and particle adhesion at the 10th hole (Figure 9a–d), developing into extensive adhesive layers, oxidative features, and thermal fatigue striations, and ultimately culminating in severe erosion pits and structural deterioration (Figure 10a–d). This progression indicates sustained thermo-mechanical overload under continuous cutting conditions. UVAD demonstrated transitional wear behavior. While significant material accumulation was observed after the 10th hole, the tool coating remained largely intact. With increasing hole count, large-scale adhesion diminished and stabilized into stratified deposits, though residual particles and localized burning persisted (Figure 11f,h), suggesting ongoing thermo-mechanical challenges. In contrast, LTUVD maintained stable tool integrity throughout the drilling sequence. Adhered layers were significantly thinner and less compact compared to UVAD, with only minor accumulation observed (Figure 11l). This improved performance stems from the synergistic combination of high-frequency impacts and torsional motion in LTUVD, which enhances chip fragmentation, reduces tool–chip contact duration, and improves heat dissipation, thereby fundamentally mitigating adhesive and thermal wear mechanisms.

In summary, tool performance progressively deteriorates with accumulated thermal degradation and adhesive wear as the number of drilled holes increases. Under continuous multi-hole machining conditions, LTUVD exhibits improved wear resistance and enhanced surface integrity compared with both CD and UVAD. It should be noted that the present assessment of coating degradation is primarily based on qualitative SEM observations. It should be noted that the assessment of coating degradation in this section is based solely on qualitative SEM observations, and quantitative measurements of coating thickness loss and delamination were not performed.

#### 3.3.2. Distribution of Tool Wear and Elemental Transfer Characteristics

**Figure 12:**
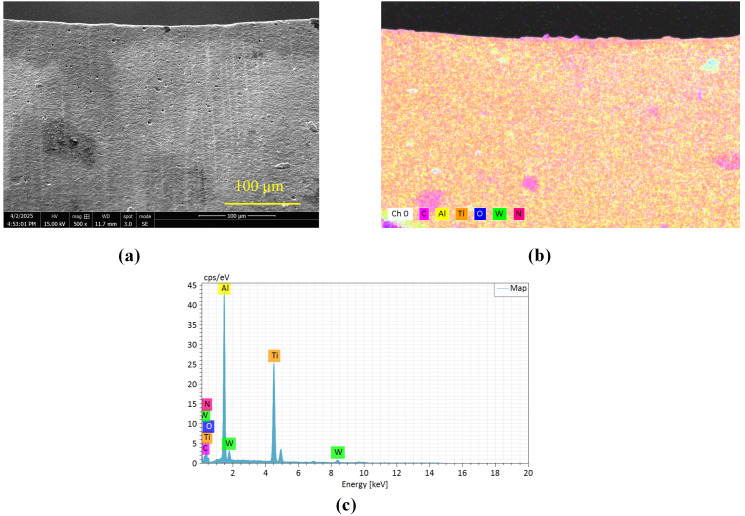
Baseline EDS analysis of an unused tool. (**a**) Morphology of the flank face at a representative location on the main cutting edge. (**b**) Corresponding EDS elemental maps, and (**c**) EDS spectrum.

To establish a baseline for elemental composition, Figure 12 presents the EDS analysis of an unused, pristine tool from the same batch. The elemental analysis targets were strategically selected to investigate interfacial material transfer mechanisms: aluminum (Al) and titanium (Ti) represent workpiece constituents, tungsten (W) and carbon (C) correspond to the WC-Co tool substrate, while nitrogen (N) and oxygen (O) derive from the TiAlN coating and oxidative wear processes, respectively. Normalized tool mass (%) values were obtained by calculating the weight percentage (wt%) of each element using the EDS software’s (Version 5.3) standard ZAF correction, then normalizing these wt% values for the selected elements (Al, Ti, W, N, O, C) to 100% to compare their relative abundance across drilling conditions and hole counts. This analytical framework enables comprehensive characterization of tribochemical interactions, coating degradation patterns, and adhesive material transfer across progressive drilling cycles. Through systematic correlation of EDS spectral data, elemental distribution profiles, and quantitative composition matrices with flank face morphology, carbon and oxygen concentrations emerge as reliable indicators for evaluating three critical wear phenomena: coating degradation, material adhesion, and thermal oxidation.

**Figure 13:**
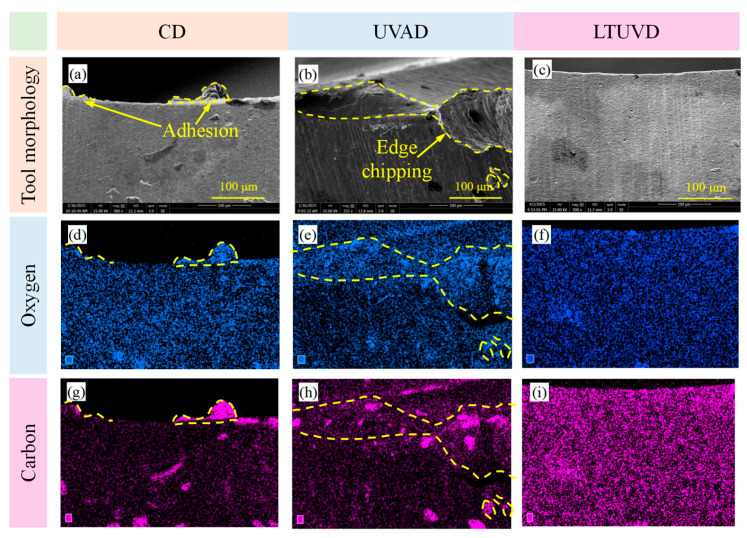
EDS analysis on the tool flank face after drilling the 10th hole. (**a**–**c**) Tool profile diagram; (**b**–**f**) Distribution of oxygen element; (**g**–**i**) Distribution of carbon element.

**Figure 14:**
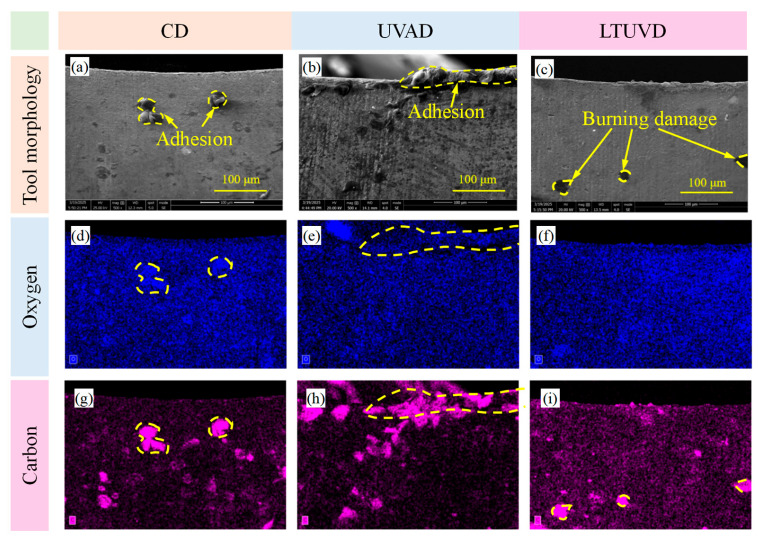
EDS analysis on the tool flank face after drilling the 20th hole. (**a**–**c**) Tool profile diagram; (**b**–**f**) Distribution of oxygen element; (**g**–**i**) Distribution of carbon element.

**Figure 15:**
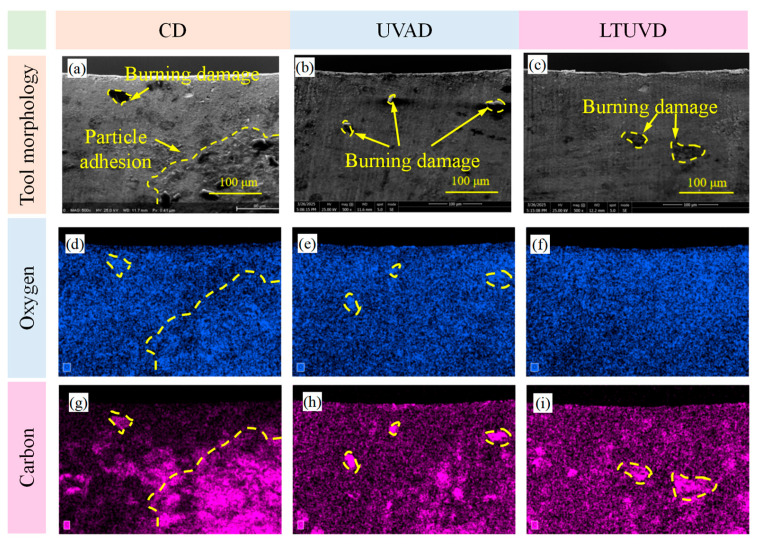
EDS analysis on the tool flank face after drilling the 30th hole. (**a**–**c**) Tool profile diagram; (**b**–**f**) Distribution of oxygen element; (**g**–**i**) Distribution of carbon element.

Figure 13, Figure 14 and Figure 15 delineate the spatial distribution of C and O across different hole counts and drilling methods, alongside the corresponding behavior of N as an indicator of coating integrity. After the 10th hole, CD shows marked carbon accumulation at the main cutting edge with reduced distribution uniformity, accompanied by a noticeable decrease in N signal intensity in the same region. This suggests that the TiAlN coating is being obscured or degraded due to adhesive material coverage and thermal exposure. UVAD reveals significant titanium enrichment in adhered layers (Table 4), confirming predominant workpiece material transfer; here, the N signal is also suppressed beneath the adhered layer, further evidencing coating coverage by foreign material. LTUVD exhibits comparatively lower oxygen content and a more stable nitrogen signal than UVAD, indicating improved adhesive effects during the initial processing stage. After the 20th hole, UVAD exhibits increased carbon accumulation at cutting edge zones, reflecting progressive thermal effects from accumulated tool wear and diminished heat suppression capacity. In contrast, LTUVD maintains uniform carbon distribution and controlled oxygen content, highlighting sustained thermal regulation during prolonged machining. Following the 30th hole, CD displays dense, uneven carbon distribution with oxygen concentration near cutting edges, indicating persistent adhesive wear and high-temperature oxidation. Both ultrasonic methods show progressively uniform carbon distribution with reduced content, confirming effective suppression of adhesive deposits. LTUVD achieves the most uniform distribution and lowest oxygen levels, indicating reduced adhesive interaction and oxidative effects, which correlates directly with reduced flank wear progression. These results conclusively establish LTUVD’s optimized vibration-assisted material interaction dynamics as the fundamental mechanism for enhanced tool integrity in multi-hole operations. 

**Table 4:** Normalized tool mass under different drilling methods and number of drilled holes.

Parameters		Normalized Mass (%)									
		Initial Tool	CD			UVAD			LTUVD		
Drill holes		-	10	20	30	10	20	30	10	20	30
Elements	Al	26.35	24.28	24.06	17.37	19.21	23.29	23.29	24.32	23.84	23.84
	Ti	29.05	24.75	22.58	17.41	41.35	24.44	24.44	29.22	26.69	24.12
	W	3.96	4.22	3.90	2.10	5.49	3.72	3.72	8.19	4.71	3.36
	N	28.24	20.68	20.68	21.17	11.05	19.23	19.23	18.20	22.18	20.89
	O	3.96	11.96	14.09	15.44	11.08	15.23	15.23	7.72	9.68	14.17
	C	7.34	14.10	14.69	26.51	11.82	14.09	14.09	12.34	12.91	13.63

**Figure 16:**
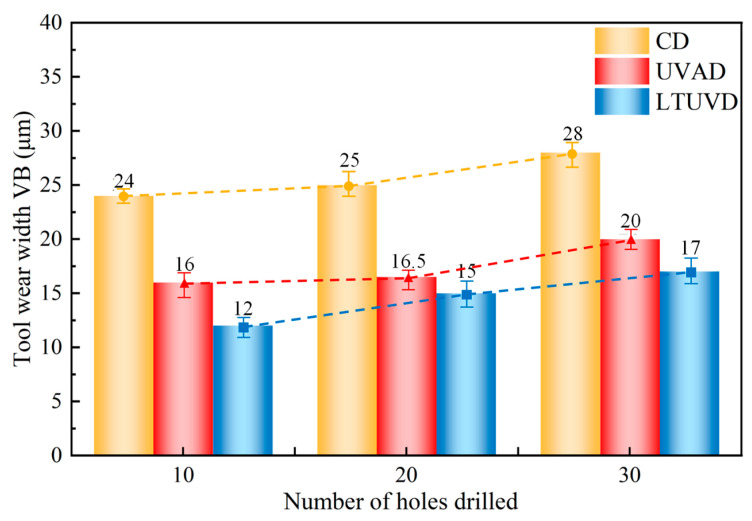
Tool wear for different drilling methods.

Flank wear width (VB) measurements under different drilling methods, summarized in Figure 16, reveal a consistent increase in VB with the number of machined holes across all techniques, though with distinct differences in severity and progression rate. Notably, LTUVD exhibits smoother and more gradual wear progression compared to CD and UVAD (Table 5). This improved performance stems from LTUVD’s inherent intermittent cutting action, reduced instantaneous uncut chip thickness, and reverse friction effects, collectively demonstrating its potential as a sustainable machining strategy.

**Table 5:** Comparison of tool wear width VB for CD, UVAD, and LTUVD at different hole numbers.

Drilling Method	Number of Holes	Tool Wear Width VB (μm)
CD	10	24 ± 0.9
	20	25 ± 2.2
	30	28 ± 1.8
UVAD	10	16 ± 2.4
	20	16.5 ± 1.5
	30	20 ± 1.5
LTUVD	10	12 ± 1.2
	20	15 ± 1.6
	30	17 ± 1.8

### 3.4. Tool Wear Effects on the Machining Quality

#### 3.4.1. Effect of Hole Number and Machining Method on Machining Quality

Quantitative evaluation of surface quality requires the introduction of 2D surface profiles and the average roughness (Ra) for supplementary analysis (Table 6). The 2D morphology provides fine details such as micro-grooves and waviness, and the Ra value offers a more quantitative and precise evaluation criterion.

**Figure 17:**
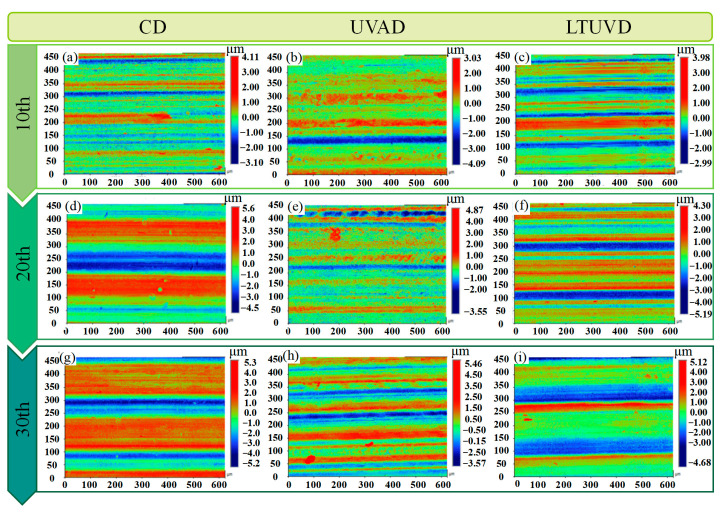
Effects of drilling method and hole number on hole wall 2D Morphology. (**a**–**c**) The surface morphology of the 10th hole; (**d**–**f**) The surface morphology of the 20th hole; (**g**–**i**) The surface morphology of the 30th hole.

Figure 17a–f shows the 2D profiles of titanium alloy hole walls after drilling the 10th, 20th, and 30th hole in the Al/Ti stack using three drilling methods, with the same machining parameters as above. Compared with the 2D profiles after drilling the 10th holes (Figure 17a–c), the wall contour in CD after drilling the 30th hole (Figure 17d) deteriorates markedly, exhibiting distinct particle indentation and scratching traces, along with noticeable material accumulation. This indicates severe tool wear and a significant loss of process stability. For UVAD (Figure 17e), the striped surface patterns become deeper and more irregular, accompanied by localized material detachment and surface disturbance, suggesting the onset of tool performance degradation. In contrast, LTUVD (Figure 17f) maintains relatively stable surface features, indicating improved hole wall quality and reduced wear progression during multi-hole drilling.

**Table 6:** Comparison of surface roughness (Ra) for CD, UVAD, and LTUVD at different hole numbers.

Drilling Method	Number of Holes	Surface Roughness Ra (μm)
CD	10	1.296 ± 0.022
	20	1.322 ± 0.061
	30	1.376 ± 0.058
UVAD	10	0.925 ± 0.066
	20	0.955 ± 0.052
	30	0.984 ± 0.050
LTUVD	10	0.844 ± 0.042
	20	0.848 ± 0.072
	30	0.879 ± 0.068

**Figure 18:**
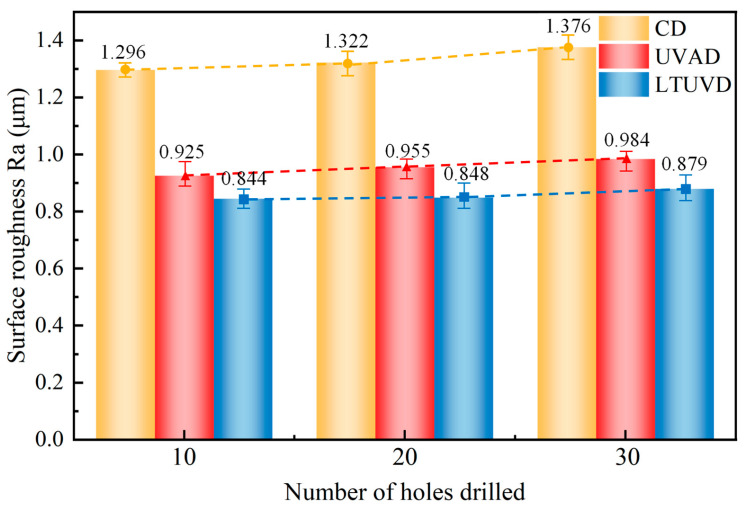
Effect of hole number and machining method on surface roughness (Ra).

Figure 18 compares the Ra values from the 10th to 30th hole under different drilling methods. Surface roughness progressively increases with cumulative hole count across all methods, though with distinct progression patterns. CD exhibits the most significant roughness escalation from an initially elevated baseline, reflecting severe process deterioration caused by progressive tool wear and built-up edge accumulation. In contrast, LTUVD maintains the lowest Ra values throughout the 30-hole sequence, indicating more stable surface roughness evolution and slower tool wear progression.

#### 3.4.2. XRD Analysis of Machining Quality

Microstructural characteristics in the hole wall region significantly influence machining quality and service performance. While previous surface topography analysis revealed parameter effects, thermo-mechanical coupling during multi-hole drilling may alter crystalline structures, affecting mechanical properties. Phase transformation, one of the predominant deformation mechanisms in metallic materials, is of particular relevance [25]. This section employs XRD to examine microstructural evolution in Ti-6Al-4V under different drilling methods, focusing specifically on α/β phase transformations.

**Figure 19:**
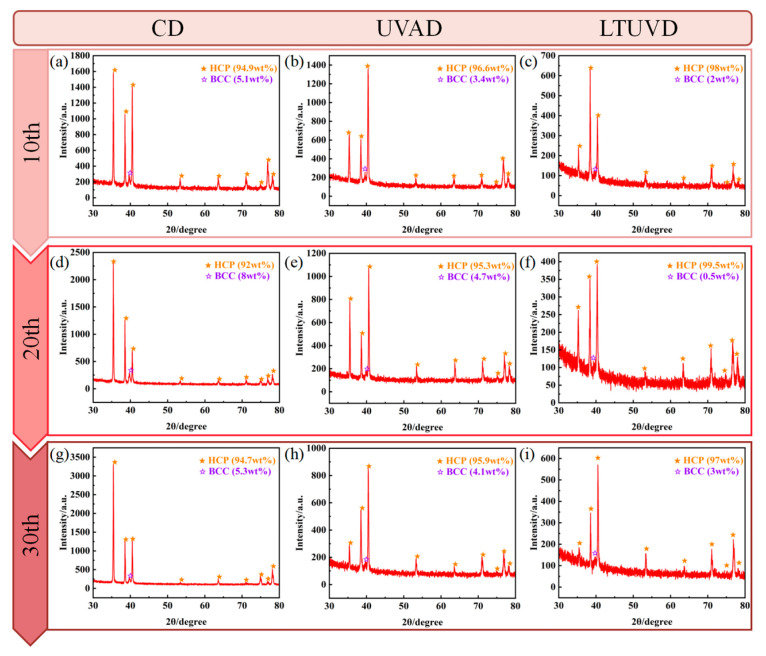
The distribution of X-ray diffraction peaks after different drilling methods and hole number. (**a**–**c**) The distribution of X-ray diffraction of the 10th hole; (**d**–**f**) The distribution of X-ray diffraction of the 20th hole; (**g**–**i**) The distribution of X-ray diffraction of the 30th hole.

XRD analysis was conducted using a Bragg–Brentano configuration with Cu-Kα radiation. Diffraction patterns were collected over a 2θ range of 30–90° with a step size of 0.02°. Phase identification was performed by matching peaks with standard reference patterns. Quantitative analysis of the α-phase (HCP) and β-phase (BCC) content was conducted using the reference intensity ratio (RIR) method, based on the integrated intensities of the α-Ti (101) and β-Ti (110) diffraction peaks, applying a standard RIR (*k_β_*/*k_α_*) value of 0.67. Figure 19 presents XRD patterns from the 10th to 30th hole drilled with each method. All patterns show dominant α-phase (HCP) characteristics with secondary β-phase (BCC) contributions. CD exhibits the highest β-phase content (approximately 18.2%), while LTUVD shows the lowest (9.8%). This progression—decreasing β-phase content from CD to UVAD to LTUVD—correlates with reduced thermal input and plastic deformation, effectively suppressing α→β phase transformation. Notably, the main α-phase peak (≈40.3°) shows negligible shift across all methods, indicating minimal residual stress introduction. Peak broadening in CD suggests grain refinement and dislocation accumulation, whereas sharper peaks in LTUVD indicate preserved microstructure integrity. Texture analysis based on relative intensities of (100), (002), and (101) planes reveals consistent orientation evolution across all methods. After 30th drilling, all methods show increased β-phase content, most prominently in CD (β-phase increase of 8.7%). Ultrasonic methods demonstrate better phase stability, with LTUVD showing the smallest increase (3.2%). The enhanced (100) peak intensity under LTUVD suggests microstructural reorientation, potentially contributing to improved mechanical stability through favorable texture development. The improved phase stability observed under LTUVD is associated with its intermittent cutting mechanism and reduced thermo-mechanical loading, which may limit microstructural evolution during multi-hole drilling.

### 3.5. Effectiveness Assessment of Drilling Methods for Al/Ti Stacks

A comparative summary of the machining performance under CD, UVAD, and LTUVD is presented in Table 7. The results clearly demonstrate the progressive improvement from CD to UVAD and, most significantly, to LTUVD across all critical indicators. LTUVD uniquely generates a spatial elliptical tool trajectory, which promotes more effective chip fragmentation, the lowest and most stable thrust forces, significantly mitigated tool wear, and the best surface integrity. This consolidated performance profile underscores LTUVD’s effectiveness as a promising technique for high-quality drilling of Al/Ti stacks. The separation of the mean values, considered together with the associated standard deviations, supports the consistency of these performance trends.

Reported LTUVD studies generally show thrust force reductions in the range of [1.98–24.9%] relative to CD, tool wear width reductions of approximately [37.31–45.15%], and surface roughness improvements of [11.61–12.32%] compared with CD under similar conditions [19,22,23]. The LTUVD approach in the present work achieves a thrust force reduction of approximately 8.51%, tool wear width reduction of 50%, and surface roughness improvement of 36.12% relative to conventional drilling under identical machining parameters. These values fall at or above the upper range of previously reported ultrasonic-assisted drilling results for LTUVD. This quantitative comparison across multiple performance metrics further supports the performance advantage of LTUVD under controlled experimental conditions.

## 4. Conclusions

This study investigated the machining performance of LTUVD in Al/Ti stacks through multi-method comparative experiments conducted at a cutting speed of 30 m/min and a feed rate of 0.1 mm/rev. The evolution of chip shape, cutting force, tool wear, and machining quality was analyzed to reveal the wear suppression mechanism of LTUVD and provide theoretical and technical guidance for high-efficiency, low-damage drilling of stack structures. The main conclusions drawn under these specific conditions are as follows:

(1) The spatial elliptical trajectory inherent to LTUVD fundamentally alters the material separation mechanism. Its intermittent cutting characteristics promote chip segmentation and prevent the formation of long, continuous chips. Improved chip fragmentation and evacuation reduce tool–chip contact duration and mitigate secondary surface damage, thereby stabilizing the drilling process from the earliest stage.
(2) The modified chip formation behavior directly governs thrust force evolution during multi-hole drilling. LTUVD maintains lower and more stable thrust forces throughout the 30-hole sequence, achieving a cumulative reduction of 18–22% relative to CD. This sustained force attenuation is associated with delayed wear-stage transition and more stable vibration transmission, demonstrating the structural robustness of LTUVD under repeated loading conditions.
(3) Compared with CD and UVAD, LTUVD presents a slower and less severe flank wear progression over 30 holes. This advantage is a direct result of its vibration-assisted cutting mechanism, which limits tool–chip adhesion and thermal loading. As verified by elemental mapping, LTUVD also best maintains tool surface integrity, with uniform carbon and minimal oxygen accumulation, proving its efficacy in mitigating the key wear mechanisms that limit tool life in multi-hole operations.
(4) The stabilization of force and wear evolution ultimately translates into improved surface integrity. LTUVD maintains the lowest surface roughness and the most stable hole wall morphology across 30 consecutive holes. Microstructural analysis further reveals the lowest β-phase content (9.8%) and minimal phase transformation increment (+3.2%), indicating effective suppression of thermo-mechanical damage accumulation in the subsurface layer.

In summary, under the investigated machining condition, LTUVD demonstrates comprehensive advantages in thrust force reduction, wear suppression, and hole-quality control for Al/Ti stacks. These findings provide a critical mechanistic benchmark and a proven solution for high-quality drilling under analogous parameters. The technology shows strong potential for application in the machining of heterogeneous laminates and precision aerospace components, although its performance envelope across a broader range of parameters warrants further investigation.

## Figures and Tables

**Table 1:** Material properties of aluminum alloy 7050-T7451 and titanium alloy Ti-6Al-4V [9].

Workpiece	7050-T7451	Ti-6Al-4V
Density (g/cm^3^)	2.70	4.43
Elastic modulus (GPa)	71	113
Ultimate tensile strength (MPa)	530	950
Yield strength (MPa)	475	900
Thermal conductivity (W/(m·K))	157	7.0
Specific heat capacity (J/(kg·K))	870	526
Coefficient of thermal expansion (×10^−6^/k)	23.6	8.6

**Table 2:** Experimental parameters.

Drilling Method	Cutting Speed (m/min)	Feed Rate (mm/rev)	Longitudinal Amplitude (μm)
CD	30	0.10	0
UVAD	30	0.10	6
LTUVD	30	0.10	6

**Table 7:** Effectiveness of various drilling methods.

Effects	CD	UVAD	LTUVD
Spatial elliptical trajectory	No	No	30 m/min, 0.10 mm/rev, 6 μm
Chip morphology	Poor	Medium	Excellent
Thrust force	High	Medium	Low
Tool wear	Poor	Medium	Excellent
Surface quality	Poor	Medium	Excellent

## Data Availability

Data are contained within the article.
